# Ionophores: Potential Use as Anticancer Drugs and Chemosensitizers

**DOI:** 10.3390/cancers10100360

**Published:** 2018-09-27

**Authors:** Vivek Kaushik, Juan Sebastian Yakisich, Anil Kumar, Neelam Azad, Anand K. V. Iyer

**Affiliations:** 1 Department of Pharmaceutical Sciences, School of Pharmacy, Hampton University, Hampton, VA 23668, USA; vivek.kaushik@hamptonu.edu (V.K.); juan.yakisich@hamptonu.edu (J.S.Y.); neelam.azad@hamptonu.edu (N.A.); 2 Great Plains Health, North Platte, NE 69101, USA; kumara@gphealth.org

**Keywords:** ionophores, chemosensitization, obatoclax, nigericin, salinomycin, combination chemotherapy, stem cells

## Abstract

Ion homeostasis is extremely important for the survival of both normal as well as neoplastic cells. The altered ion homeostasis found in cancer cells prompted the investigation of several ionophores as potential anticancer agents. Few ionophores, such as Salinomycin, Nigericin and Obatoclax, have demonstrated potent anticancer activities against cancer stem-like cells that are considered highly resistant to chemotherapy and responsible for tumor relapse. The preclinical success of these compounds in in vitro and in vivo models have not been translated into clinical trials. At present, phase I/II clinical trials demonstrated limited benefit of Obatoclax alone or in combination with other anticancer drugs. However, future development in targeted drug delivery may be useful to improve the efficacy of these compounds. Alternatively, these compounds may be used as leading molecules for the development of less toxic derivatives.

## 1. Introduction

### Ion Transport

Ion homeostasis is extremely important for the survival of cells. A proper balance of ions both inside the cells as well as in the extracellular matrix is necessary for the maintenance of membrane potential, cell shape and proper functioning of several cellular pathways. Cell membrane consists of two lipid bi-layers where polar heads of the lipids face outward and hydrophobic tails form interior of the cell membrane. This structural composition of plasma membrane results in impermeability of various ions, small hydrophilic molecules such as glucose and macromolecules such as proteins and RNA across the cell membrane. Only water, oxygen and carbon dioxide freely move across plasma membrane. Cells overcome these transport issues by devising mechanisms for facilitated diffusion as well as active transport of ions and molecules across membrane. Facilitated transport involves diffusion of ions towards concentration gradient mediated by proteins which form water filled ion channels across the membrane. These ion channels are gated and can be opened and closed based on the cellular requirements. The most common types of gated ion channels are ligand-gated, mechanically gated, voltage-gated, and light-gated [1]. In active transport ions or molecules are transported against the concentration gradient with the help of transporter proteins using energy from the ATP. Na^+^/K^+^ ATPase, H^+^/K^+^ ATPase, Ca^2+^ ATPase, ABC transporters are a few examples of active transporters [2]. Aberrant expression and/or functioning of ion channels and ion pumps in cancer cells establish a unique ion homeostasis which is specifically advantageous to cancer cells. Maintenance of this ion homeostasis is of great interest to cancer cells. In following sections of the review article, we will discuss how cancer cells maintain a particular ion balance and how it helps them in escaping death, increased cancer cell proliferation, and metastasis. We will also explore how this ion homeostasis is targeted to develop novel therapeutic interventions for cancer treatment and discuss role of ionophores as anticancer drugs. Ionophores are a class of compounds which have been successfully employed to eliminate cancer by manipulating ion balance in cancer. We will focus on Salinomycin (SAL), Nigericin (NIG) and Obatoclax (OBT) as these ionophores have shown well-documented potent anticancer activity against cancer stem-like cells as well as promising use as chemosensitizer. Although other ionophores have shown anticancer activity and some of them may be active against putative cancer stem cells (CSCs) the information is limited or they are less promising than SAL, NIG or OBT. For instance, Valinomycin’s activity against cancer cells were less potent compared to SAL or NIG [3]. (Table 1) We will also track clinical progress of these drugs by evaluating various clinical trials exploiting these drugs as potential anticancer agents.

## 2. Ion Transport in Cancer Cells and Its Targeting to Develop Novel Anticancer Therapies

### 2.1. Ion Transport in Cancer Cells

Growing body of evidence suggest an altered ion transport in cancer cells. Cancer cells rewire their cellular circuitry to establish, adopt, proliferate, and metastasize in various challenging conditions by manipulation their ion homeostasis and ion channels and ion pumps play a critical role in this reorganization [30].

Ca^2+^ is a very important ion and plays key role in various signaling mechanism, which integrate with other signal-transduction cascades and controls a variety of cellular processes. Therefore, intracellular Ca^2+^ (Ca^2+^i) concentration is precisely maintained for proper functioning of cells. Ca^2+^ homeostasis is maintained by calcium permeable channels such as transient receptor potential (TRP) channels, store-operated channels (SOCs), voltage-gated calcium channels, as well as mitochondrial calcium uniporter (MCU), voltage-dependent anion channels (VDACs), IP_3_ and ryanodine receptors, and others. Ca^2+^i plays central role in early G1phase and at the G1/S and G2/M transitions [31]. Other studies have pointed towards Ca^2+^/calmodulin (CaM) and Ca^2+^/calcineurin pathways as major checkpoints in cell cycle progression [32,33]. Metastasis involves several Ca^2+^ dependent processes, including cell deformation, invasion, migration, and adhesion. TRPM7 channels have been shown to form local and transient calcium domains known as “calcium flickers” at lamellipodia and guide the direction of migration [34]. An in-depth account of Ca^2+^ transport mediated proliferation and metastasis in prostate, breast and lung cancer can be found in a review article by Deliot et al. [35] Apoptosis involving calcium ion overload in cytosol is a well explored process. Apoptotic cells increase intrinsic Ca^2+^ either by sustained Ca^2+^ influx via activated channels or release of calcium by a stressed endoplasmic reticulum (ER) [36,37]. Cancer cells exhibit greater apoptotic resistance by inhibiting calcium influx by down-regulating channels and/or adapt to chronic-reduced ER Ca^2+^ [36].

K^+^ is the most predominant ion inside the cell and its cellular concentration is maintained by four different classes of ion channels―voltage-gated, calcium-activated, inward rectifier and two-pore-domain potassium channels. K^+^ ion is key regulator of cell volume and shrinking of cell volume is a characteristic phenotypic modulation of cells undergoing apoptosis. Loss of intracellular K^+^ via activation of various ion channels leads to apoptosis by decay of the membrane potential and the associated Ca^2+^ influx, apoptotic volume decrease, and activation of various enzymes involved in the apoptotic process [38]. Readers can refer to a review by Wang for the potential role played by K^+^ ion channels in cancer cell proliferation and apoptosis [39]. Similarly, Na^+^ and Cl^−^ ion and ion channels have been associated with cancer proliferation and resistance to apoptosis [38,40]. Zn^2+^ transport has been shown to affect epithelial mesenchymal transition (EMT) and metastatic stature of cancer [41]. Zn^2+^ accumulation has been linked to increased resistance as well as sensitization of cancer in cell dependent manner [42,43]. Mg^2+^ homeostasis is often associated with drug resistance of cancer cells [44,45]. Cu^+^ is essential micro nutrient and its levels are maintained by several transporter and chaperone proteins. Cu^+^ is selectively taken up by Copper Transport Protein 1 (CTR1) and then distributed inside the cell to various cellular compartments by three chaperone proteins Atox1, Cox17p and CCS. While Cox17p and CCS transport Cu^+^ to mitochondria and copper/zinc superoxide dismutase respectively; Atox1 carries Cu^+^ to ATPase transporters ATP7A and ATP7B which move Cu^+^ to trans-golgi network or secretory vesicles for copper efflux from the cells [46]. Pt^2+^ exhibits similar coordination chemistry that of Cu^+^ and highjack copper transport machinery for transport of platinum-based anticancer drugs. Modulation of expression and activity of CTR1, ATP7A and ATP7B has been shown to impart resistance to platinum-based chemotherapies by reducing their cellular levels either by inhibiting their uptake or increased efflux of internalized drug respectively [47,48,49,50].

Cancer cells switch their metabolism to glycolysis to meet their energy requirements, known as Warburg effect. This metabolic change leads to accumulation of lactate in the cells. This excess lactate is pumped outside the cell by the over activation of Na^+^/H^+^ exchanger 1 (NHE1) and the H^+^/lactate cotransporter in cancer cells making tumor microenvironment highly acidic. This deregulated pH homeostasis results in cellular alkalization which was suggested to be first step in the carcinogenesis [51]. Cancer cells use this acidic environment to their advantage as they are more adept to these conditions than normal cells. Furthermore, this acidic environment shields cancer from weakly basic drugs by protonating them resulting in their decreased partitioning inside cancer cells [52]. Alternatively, H^+^ ion channels and pumps play a key role in malignant transformation of cancer by inducing metastasis. Cancer metastasis involves cell volume and cell shape modifications as cell develops lamellipodium and invadopodium to migrate and invade through extracellular matrix. These changes in cell volume are regulated by local ion transport through the ion channels at the leading edge and tip of the outgrowing lamellipodium/invadopodium. Several studies have pointed towards a critical role played by NHE1 in tumor progression and invasion [53]. H^+^ ion channels also play important role in cell signaling, proliferation and cell cycle.

### 2.2. Ion Transport and Chemotherapy

Modulation and maintenance of altered ionic homeostasis by cancer cells is well documented. Therefore, alteration of this ionic balance may serve as a lynch pin to start a cascade of signaling events ultimately leading to cancer cell death. There is multitude of evidence to support this notion as several studies indicated modulation of cellular ion homeostasis by either activation or deactivation of ion transporters and ion channels sensitizes cancer cells to otherwise in effective drugs. Regarding their potential use as chemosensitizers, Abdoul-Azize et al. recently demonstrated sensitization of pediatric acute myloid leukemia to dexamethasone by chelation of intracellular Ca^2+^ ions with calcium chelator BAPTA-AM [54]. Similarly, phenyl isothiocyante induced apoptosis in Gefitinib-resistant NCI-H460 human lung cancer cells by altering Ca^2+^i levels [55]. Chen et al. reported reversal of chemoresistance to platinum drugs in oxaliplatin-resistant human cervical cancer cells in vitro and in vivo in response to co-treatment with iron chelator desferal [56]. Partial restoration of function of volume-sensitive outwardly rectifying (VSOR) chloride channels in cisplatin resistant KCP-4 human epidermoid cancer cell line by treatment with trichostatin A sensitized these cells to cisplatin [57]. Esomeprazole a proton pump inhibitor potentiated antitumor activity of doxorubicin in triple negative MDA-MB-468 breast cancer cells [58]. Specific ion pump inhibitors have been tested for cancer treatment. For example, cardiac glycosides which are Na^+^/K^+^ pump inhibitors and commonly used for heart ailments are currently being investigated for their anticancer properties in various cancer. Several studies have pointed towards their potential anticancer efficacy in different types of cancers [59,60,61,62]. Similarly, hERG1 inhibitors, which block kv11.1 channels, have shown remarkable antitumor activity in several cancers [63,64]. Even though, ion channel/ion pump inhibitors have demonstrated potent anticancer activity, their use as anticancer drugs is limited due to limited selectivity. For instance, cardiac glycosides have very narrow therapeutic index (low nano molar range) as at higher concentrations they exhibit severe cardio toxic side effects. Nonspecific inhibition of kv11.1 channels by hERG1 inhibitors may also lead to the lengthening of the electrocardiographic QT interval, thus predisposing the patient to ventricular arrhythmias [65]. Ion transport inhibitors may have other effects that can be exploited as chemosensitizer. For instance, the Ca^++^ channel inhibitor Verapamil is also a potent inhibitor of the multidrug resistant (MDR) protein ABCB1/P-glycoprotein (P-gp) and this property has been tested to overcome multidrug resistance [66]. Since the P-glycoprotein, can be modulated by calcium channel blockers such as cyclosporin and nifedipine these inhibitors were able to reverse drug resistance in tumors [67,68]. However, at present the use of calcium channel blockers in chemotherapy has not been successfully translated into clinics, perhaps to limited availability and toxicity. For instance, while the average steady-state plasma levels measured for verapamil is ~0.5 μM the concentration usually associated with MDR1 inhibition is about ≥50 µM [66] indicating that this drug may not be useful at the clinical level.

Therefore new, more specific and selective ion transport modulators are needed to effectively exploit cancer’s ionic homeostasis as a target to develop successful therapy interventions against cancer.

## 3. Ionophores

**Figure 1:**
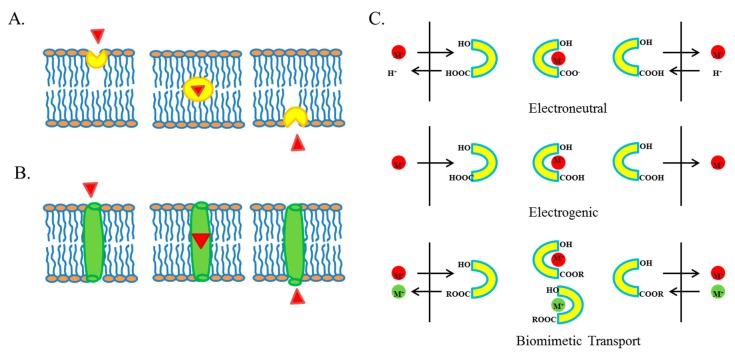
Ionophore mediated ion transport across the membrane. (**A**) Small ionophores “ion carriers” bind with ion, shield it from lipophilic interior of membrane, transport it across the membrane and release it other side of membrane. (**B**) Large ionophores form “ion channels” across the membrane and transport ions through these channels. These channels have a hydrophilic interior which assist in transport of ions while its lipophilic exterior shield ions from repulsive interior of membrane. (**C**) Polyether ionophores carry ions across membrane by electroneutral, electrogenic and biomimetic methods based on the microenvironmental conditions and structure of ionophore. Panel C was modified from [71].

Ionophores mean “ion carrier” is a class of compounds which can bind non-covalently with ions and can assist in their transport across the cell membrane. Ionophore consists of lipophilic exterior and has a hydrophilic interior where ion binds and transported across lipid membrane. Ionophores can be divided in two categories depending on the size of the molecule that in turn determines the mode of transport of the ion across the cell membrane (Figure 1). Small ionophores such as valinomycin form complex with the ions and transport them across the cell membrane are known as “Ion Carriers” [69]. Small polyether ionophores transport ions by electroneutral, electrogenic or biomimetic mechanisms depending on micro environmental and structural modification of ionophore [70,71,72]. Large ionophores such as gramicidin form channels across the cell membrane for the ion transport. The interior of channel is hydrophilic and assists in ion transport while lipophilic external shell shields ion from hydrophobic environment of cell membrane [69]. However, ion channel ionophores exhibit a lower selectivity in comparison with ion carrier ionophores. Based on chemical structure ionophores can be categorized as polyether, peptide, cyclodepsipeptide, macrotetrolides and cryptates [69]. Polyether ionophores consist of an oxygen rich hydrophilic interior which mostly comprises of ether bonds. Salinomycin and nigericin are example of polyether ionophores. Gramicidin is example of peptide ionophores where peptide bonds contribute towards ionophortic activity of the peptide. Cyclodepsipeptides are peptides in which one or more peptide bonds are replaced by ester bonds. Enniantin is an example of this class of ionophores. Nonactin is a macrotetrolide and has a macrocyclic structure comprising of four tetrahydrofuranyl-ester residues. Commonly, ionophores have been used as anticoccidial drugs for poultry and/or growth promoters in ruminants. Lately, ionophores have found a new role as anticancer drugs due to their ability to alter this ion balance by ion transport across cell membrane.

### 3.1. Salinomycin (SAL)

SAL is a naturally occurring polyether antibiotic [73] isolated from *Streptomyces albus* strain. It has been used as antibacterial and coccidiostat. SAL is an ionophore and imparts its antibacterial properties by facilitating transport of K^+^ ions through the cell membrane of target organism leading to an increase of intracellular Ca^2+^ ions. This disruption of ionic homeostasis leads to deregulation of osmotic balance resulting in death of the organism. SAL came in forefront as a potential anticancer drug in 2009 when Gupta et al. screened roughly 16,000 compounds for their selective anticancer efficacy against CSCs and found SAL at least 100-fold more effective than paclitaxel, a commonly used anticancer drug [7]. Following this work, several other studies pointed towards SAL’s selectivity in targeting cancer stem cells practically in every type of cancer [74,75,76,77,78,79,80] as well as other multidrug resistance (MDR) cancer cells [81,82]. Although the exact mechanism by which SAL targets cancers is not known, it is very clear that it influences multiple pathways to impart its effects. SAL has been shown to induce cancer and CSC death by inducing apoptosis [83,84,85,86,87,88]. There are studies which indicate autophagic cancer cell death by SAL [89,90]. However, several studies present a contradictory view as they suggest inhibition of autophagy and induction of apoptosis as the mechanism for elimination of cancer by SAL [91,92,93,94,95]. Several studies indicated SAL induced oxidative stress as a key mediator for apoptotic cell death [84,96,97,98]. SAL induces oxidative stress by altering mitochondrial membrane potential. Beside its specific cytotoxicity towards cancer and CSCs SAL regulates cancer metastasis by inhibiting cancer cell invasion and migration by targeting Wnt and EMT pathways [78,99,100,101,102]. Several studies indicate possible involvement of Hedgehog signaling in SAL induced cell death in breast cancer [103,104]. SAL is a potent partner in a co-therapy approach and has been shown to sensitize several cancers and to potentiate efficacy of other commonly used anticancer drugs such as doxorubicin, trastuzumab, gemcitabine, tamoxifen etc. [105,106,107,108] Zhang et al. demonstrated SAL induced sensitization of pancreatic cancer to gemcitabine by targeting CSCs [108]. In a recent study Venkatadri et al., observed a sensitizing effect of SAL in breast cancer where SAL was able to potentiate the resveratrol’s anticancer effect at low concentration which was rather ineffective when used independently [109].

Cancer resistance is a major concern in complete eradication of cancer as some of the cancer cells acquire or exhibit resistance to therapy and escape elimination. These cells come back as a more aggressive and more resistance cancer and cause a cancer relapse. Most of the current therapeutic approaches fall short of achieving complete cure of cancer and after initial remission cancer relapses in several cases. Interestingly, SAL has demonstrated potent anticancer effect in various multi drug resistant (MDR) cancers [110,111,112]. Development of drug efflux mechanisms via various drug transporter proteins such as p-glycoproteins, ABCG, MDR etc. is the most commonly employed counter measure by cancer to survive a fatal outcome. SAL has been shown to overcome drug resistance by inhibiting these drug transporters [105,113,114].

SAL has exhibited a great therapeutic potential as an anticancer drug. However, poor water solubility and toxicity to normal cells is a major concern in its therapeutic application for cancer treatment. These issues can be addressed by either development of targeted delivery strategies and/or by synthesis of less toxic and more specific SAL analogues. Recently, several studies have reported successful use of nanoparticles, nanomicelles, nanotubes, and multilamellar liposomes conjugated with cancer cell surface markers such as CD133, CD44 etc. for targeted delivery of salinomycin [115,116,117,118,119]. Many analogues have been synthesized by introducing different substituent, functional groups in the core structure of salinomycin and have been tested for their anticancer efficacy [120,121]. SAL demonstrated unique ability to target CSCs and MDR cancer cells in variety of cancers bestowing it with enormous potential to be a break through drug as a mono therapy or as a sensitizer to compound the effects of other anticancer agents in resistant cancers.

### 3.2. Nigericin (NIG)

NIG is an antibiotic derived from Streptomyces Hygroscopicus. It was first isolated by Harned et al. from an unidentified Streptomyces which was later reported to be *Streptomyces “Nig-1”* [122,123]. NIG is an ionophore and can transport K^+^, H^+^ and Pb^2+^ ions across the plasma membrane. NIG is an antiporter of H^+^ and reduces internal pH (pHi) of cells. Cell proliferation is a pH sensitive process and DNA replication requires slightly alkaline pH to perform optimally. Several studies have indicated stimulation of cell growth resulting from rapid increase of pHi (0.1–0.3 pH units) in response to addition of growth promoting reagents such as serum and growth factors [124]. This increase in pHi in response to growth factors was mediated by stimulation of amiloride sensitive Na^+^/H^+^ exchange [125]. Cancer cells have a reversed pH gradient then normal cells which have pHe > pHi while cancer cell maintain a pHe < pHi. This reversed pH gradient is advantageous to cancer cells as it promotes cancer progression by inducing cancer invasion and migration [126]. NIG has shown potent anticancer potential in several cancers as well as resistant cancer stem cells [5,6]. Exact mechanism by which nigericin acts is not known but several studies have pointed towards its ability to antiport H^+^ inside the cell as possible mode for its anticancer activity. Cancer cells have an acidic external pH (pHe) and nigericin exploits these external cancer microenvironment conditions to its advantage by transporting H^+^ from exterior to interior of the cell resulting in lowering of pHi which in turn leads to cancer cell death. Margolis et al. reported a decrease in cytoplasm pH and reduction in DNA synthesis on treatment with millimolar concentrations of NIG in Ehrlich ascites tumor cells [127]. Alteration of mitochondrial function has been associated with anticancer activity of NIG. Treatment with NIG alters membrane potential which may lead to disruption of energy balance and/or increased reactive oxygen species (ROS) production leading to cancer cell death [3,128].

Photodynamic therapy (PDT) is a form of phototherapy which is minimally invasive and least toxic. It employs a light source which excites the photo sensitizer which in turn interacts with molecular oxygen to produces radicals and ROS leading to localized cytotoxicity in the exposed tissue. Several studies have pointed towards NIG mediated sensitization of cancer cells for photodynamic therapy [129,130]. There is no clear mechanistic explanation for this synergistic effect as evidence suggest change of pHi, inhibition of peroxidase detoxification to translocation of Bax to mitochondria as possible reasons for this outcome [131,132]. In another study, Varnes et al. observed an inhibition of potentially lethal damage recovery in A549 cells upon treatment with micro molar concentration of NIG post PDT [133]. NIG sensitizes cancer cells and demonstrated a synergistic effect with other anticancer drugs leading to an improved efficacy of these drugs [6,134]. This enhanced efficacy was mostly related to NIG’s ability to reduce pHi under acidic tumor microenvironment [134,135]. Regulation of key cellular pathways such as EMT and Wnt/β catenin has been shown to induce cytotoxicity and inhibition of cancer metastasis on NIG treatment [136,137]. Autophagy inhibition potentiates NIG treatment. In a recent study Vu et al. demonstrated a reduction in spheroid formation by ATG5 deficient glioma cells on treatment with NIG [138].

### 3.3. Obatoclax (OBT)

OBT is synthetic derivative of Prodigiosin class of compounds. Prodigiosin are red pigments produced by bacteria and show antimalarial, antifungal, immunosuppressant, antibiotic, and anticancer properties. OBT is a BH3-mimitic and induces apoptosis by modulating Bcl2 family proteins. It has shown cytotoxicity in several cancers by inducing apoptotic cell death.

Often faced with harsh conditions such as nutrient deficiency, hypoxia, DNA damage, unnatural genetic variation, and instability, cancer cells still manage to survive by evading death signals by modulating pro-survival regulators. One such survival design includes over expression of anti-apoptotic Bcl2 family proteins. These proteins antagonize pro-death proteins by forming a heterodimer via binding to the pro-apoptotic protein’s BH3 domain situated in the hydrophobic cleft of anti-apoptotic proteins. While pro-death proteins have only BH3 homology domain, Bcl2 proteins have four homology domains (BH1-4). Other apoptotic proteins Bax and Bak which are necessary for the cell death by BH3 only proteins share three homology domains (BH1-3). In viable cells, Bax and Bak exist as monomers; however, they form homooligomers upon activation of BH3-only pro-apoptotic proteins. These homooligomers insert themselves in mitochondrial membrane and permeabilize it for the release of cytochrome c and other pro-apoptotic factors needed for mitochondria mediated apoptotic cell death. Therefore, natural or synthetic BH3 mimics represent a promising therapeutic approach for targeting cancer cells.

OBT’s anticancer potential is often attributed to its BH3 targeted apoptosis induction in cancer cells. In a recent study, Díaz de Greñu et al. demonstrated ionophoretic activity of OBT and other synthetic derivatives towards chloride and bicarbonate anions and correlated cytotoxicity of these compounds for small-cell lung carcinoma cell line GLC4 with their ability to discharge pH gradient in living cells [139]. Acridine orange (AO) a membrane permeable dye which accumulates in acidic vesicles such as lysosomes was used to monitor pH changes with OBT treatment. AO have a characteristic orange fluorescence at acidic pH while it flourishes green at basic pH. A transition from orange to green fluorescence for AO was observed upon treatment with OBT indicating a possible bicarbonate ion transport leading to alkalization of lysosomes by OBT.

Even though apoptotic cell death constitutes the primary mode of cytotoxicity of OBT in cancer cells, other cell death mechanisms such as autophagy, necrosis, necroptosis etc. have been reported as possible causes of cancer cell elimination further corroborating existence of an alternative mechanism of drug activity. There are several reports which suggest autophagy as possible effecter of cell death [140,141]. Obatoclax can induce apoptosis in Beclin 1 dependent and independent manner [142,143,144,145]. There is evidence that suggests autophagy and apoptosis work in tandem to eliminate cancer cells [146]; however, there are contradictory reports that suggest obatoclax inhibits autophagy by impairing lysosomal function to induce cytotoxic effects in cancer [147,148]. In a recent study, Basit et al. reported necroptosis mediated cell death by induction of fusion of necrosome on autophagosomal membrane on treatment with obatoclax [149]. Champa et al. observed necrotic cell death in highly resistant anaplastic thyroid cancer cells upon treatment with OBT. On treatment OBT quickly localized in lysosomes and neutralized their pH. Interestingly, OBT activity was dependent on its accumulation in lysosome rather than its interaction with Bcl2 family proteins. OBT has demonstrated a potentiating effect on cancers in combination with other chemotherapeutics [150,151]. Li et al. reported a synergistic effect between OBT and cisplatin in non-small-cell lung cancer [152]. Resistance to therapy is a major concern in cancer treatment. OBT has shown great response in resistant cancer cells and has overcome chemoresistance in different cancers [153,154].

OBT has shown significant anticancer activity in leukemia cells which rely on Bcl2 family proteins for their survival. OBT has demonstrated specificity towards leukemia cancer stem cells [14]. Several studies have identified OBT as potential chemotherapeutic agent to overcome glucocorticoid resistance in leukemia [155,156]. Wei et al. revealed synergistic antileukemia activity of OBT with a histone deacetylase inhibitor [146]. OBT’s promising preclinical efficacy has not translated in clinical trials. Phase I studies of OBT in chronic lymphocytic leukemia (CLL) patients demonstrated significant toxicities such as somnolence, ataxia, and confusion with limited efficacy [157].

It is indisputable that OBT is a BH3 inhibitor; however, there is ample evidence that suggest an alternate mechanistic intervention for OBT’s anticancer activity. Furthermore, several studies indicated neutralization or alkalization of lysosomes upon treatment with OBT as the leading event in OBT induced cancer cell death suggesting ionophoretic activity of OBT playing a prominent role in anticancer activity of the drug. Also, OBT’s specific activity towards CSCs and its ability to sensitize resistant cancers indicates immense anticancer potential of OBT. It is imperative to understand various molecular targets and pathways involved in OBT induced therapeutic effects. OBT can be a lucrative therapeutic intervention for cancer sans its toxic side effects and perhaps a better understanding of its activity will render it more clinical relevance.

## 4. Ionophores in Ongoing Clinical Trials for Cancer Treatment

There are a few animal studies demonstrating that NIG, at tolerated doses, in combination with other compounds has antitumor effects [158]. However, at present there are no registered clinical trials using NIG as anticancer agents www.clinicaltrials.gov.

There are a few clinical reports in the literature about the use of SAL that comes from pilot studies involving a few patients (four metastatic breast cancer patients, a metastatic ovarian cancer patient, and a patient with head and neck squamous cell carcinoma) [9,159]. At present there are no registered clinical trials at www.clinicaltrials.gov.

OBT has been tested in multiple clinical trials as single agent as well as in combination with other anticancer agents (Table 2). As single agent OBT was not associated with an objective response in AML [160] or showed only a modest activity in heavily pretreated patients with advanced CLL [157], classic Hodgkin lymphoma (cHL) [161] myelodysplastic syndromes [162]. OBT in combination with carboplatin/etoposide failed to significantly improve objective response rate (ORR), progression-free survival (PFS) or overall survival (OS) in first-line treatment of extensive-stage small cell lung cancer (ES-SCLC) [163]. Similarly, when added to topotecan did not exceed the historic response rate seen with topotecan alone in patients with relapsed SCLC following the first-line platinum-based therapy [164].

There are several factors that may limit the clinical translation of ionophores as anticancer agent especially as single agents. On one hand, the pharmaceutical industry may be more interested in pursuing patentable drugs. However, toxicity may be the main concern. For instance, SAL is very toxic to other normal cells at concentrations effective against cancer stem-like cells [166] and therefore it is unlikely that this drug will be useful as single agent [167]. The ability of NIG to induce apoptosis or necrosis by increase K^+^ efflux occurs only at relatively high concentration (2.5–7.5 µM) [6] that may be not tolerated in vivo. However, as previously discussed, other biological effects at tolerated doses may be exploited for combination therapy in clinical trials.

## 5. Conclusions

The disruption of ion homeostasis important for proliferation and survival of cancer constitutes a potential target for chemotherapy. The ionophores SAL, NIG and OBT have shown important anticancer activities in in vitro and in vivo preclinical models of cancer as single agents as well as in combination with other anticancer drugs. More important, they also showed anticancer activity against putative cancer stem-like cells. The underlying reason some ionophores work against cancer stem cells and other ionophores do not is poorly understood but it is possible that other ionophore-independent activity target key processes associated with stemness, for instance, (i) SAL induces ER Ca^2+^ depletion up-regulating C/EBP homologous protein (CHOP), which inhibits Wnt signaling by down-regulating β-catenin [168]. SAL also inhibits K-ras [169], Notch [82] and Hedgehog signaling [170], (ii) NIG is also a potent modulator of the Wnt signaling pathway [6] and (iii) OBT targets cancer stem cells via disruption of BCL-2-dependent oxidative phosphorylation [14].

Except for OBT, these ionophores have not been translated into clinical trials. At present, the results of clinical trials with OBT as single agent or in combination with other anticancer drugs did not show a significant benefit. It is possible that the high toxicity of ionophores towards non-cancer cells may be limiting their clinical use. The selectivity towards non-cancer cells can be investigated by using non-cancer cell lines from the same organ. For instance, the Beas-2B cell line consists of epithelial cells that were isolated from normal human bronchial epithelium obtained from autopsy of non-cancerous individuals and is sometimes used to compare to lung cancer cells [171]. To overcome this limitation, future development in targeted drug delivery may help to improve the ability of these promising compounds. The use of different types SAL loaded nanoparticles alone or in combination with other drugs showed improved efficacy compared to SAL alone in a variety of cancer cell types (Table 3). Alternatively, SAL, NIG and OBT may serve as lead compounds to develop derivatives more selective towards non-cancer cells. In this context, several derivatives of OBT [172,173] and SAL [120,121,174,175,176,177,178,179] have shown anticancer effects. For instance, derivatives with chemical modification of the allylic C20 hydroxyl of SAL, located at the C-ring, enhanced the activity over 5-fold against breast cancer cells compared to the native structure [121]. Derivatives of OBT were also found to be more potent against PLC5 hepatocellular carcinoma cells than the original compound [173]. At present the biological effects and selectivity, in particular the ability to deplete chemoresistant cells such as cancer stem-like cells need to be further investigated in more advanced preclinical (animal) models. In summary, targeted delivery and development of more potent and selective synthetic derivatives of concerned ionophores can facilitate the translation into clinical applications for cancer treatment.

## Figures and Tables

**Table 1:** Ionophores with anticancer activity.

Ionophore	Transported Ion	Cancer Type	Target CSCs	Reference
Nigericin	K^+^, H^+^	Several	Yes	[4,5,6]
Salinomycin	K^+^, Ca^2+^	Several	Yes	[7,8,9]
Obatoclax	HCO_3_^−^, Cl^−^	Several	Yes	[10,11,12,13,14,15]
Gramicidin	H^+^, Na^+^, K^+^	Renal cell carcinoma,	Not known	[16,17]
Ionomycin	Ca^2+^	Breast	Not known	[18,19]
Monensin	Na^+^, H^+^	Glioblastoma, Bladder	Not known	[20,21,22]
Valinomycin	K^+^	Ovarian, Colorectal,	Likely	[3,23,24]
Lasalocid	K^+^, Na^+^, Ca^2+^,	Prostate	Not known	[25]
Enniatin	Mg^2+^	Colon, Ovarian	Not known	[26,27]
Beauvericin	NH_4_^+^, Ca^2+^, Ba^2+^	Prostate Cervical, Colorectal Hepatoma, Lung	Not known	[28,29]

**Table 2:** Registered clinical trials (www.clinicaltrials.gov) and published results.

Ionophore	Condition	Other Drugs	Phase	Clinical Trial	Published Results
Salinomycin	-				
Nigericin	-				
Obatoclax	AML	-		NCT00684918	[160]
	Chronic Lymphocytic Leukemia	-	I/II	NCT00600964	[157]
	Extensive-stage Small-Cell Lung Cancer	Carboplatin/etoposide	I/II	NCT00682981	[163]
	Lymphoma, Follicular	Rituximab		NCT00427856	-
	Non-Hodgkin Lymphoma Recurrent Adult Diffuse Large Cell Lymphoma Recurrent Grade 1 Follicular Lymphoma (and 5 more…)	Bortezomib		NCT00538187	-
	B-cell Chronic Lymphocytic Leukemia Leukemia Prolymphocytic Leukemia (and 5 more…)	Fludarabine rituximab		NCT00612612	-
	Leukemia Systemic Mastocytosis			NCT00918931	-
	Mantle-Cell Lymphoma	Bortezomib		NCT00407303	-
	Hodgkin’s Lymphoma			NCT00359892	[161]
	Extensive-stage Small-Cell Lung Cancer	Carboplatine and Etoposide		NCT01563601	-
	Lung Cancer	Docetaxel		NCT00405951	-
	Myelodysplastic Syndromes			NCT00413114	[162]
	Acute Leukemias of Ambiguous Lineage Acute Undifferentiated Leukemia Angioimmunoblastic T-cell Lymphoma (and 26 more…)	Dexrazoxane hydrochloride Doxorubicin hydrochloride (and 3 more…)		NCT00933985	[165]
	Refractory Multiple Myeloma Stage I Multiple Myeloma Stage II Multiple Myeloma Stage III Multiple Myeloma	Bortezomib		NCT00719901	-
	Recurrent Small-Cell Lung Cancer Unspecified Adult Solid Tumor	Topotecan hydrochloride		NCT00521144	[164]
	Extranodal Marginal Zone B-cell Lymphoma of Mucosa-associated Lymphoid Tissue Nodal Marginal Zone B-cell Lymphoma Recurrent Grade 1 Follicular Lymphoma (and 4 more…)	Bendamustine hydrochloride		NCT01238146	-
	Myelofibrosis			NCT00360035	-
	Hematological Malignancies			NCT00438178	-
	Leukemia (samples)			NCT01150656	-
	Metastatic Melanoma	Temozolomide	I/II	NCT00724841	-

**Table 3:** Delivery of Salinomycin using nanoparticles.

Nanoparticle	Cancer Type	Efficacy	Reference
SS lipid-polymer hybrid nanoparticles	Lung	↑	[180]
CESP *	Osteosarcoma	↑	[181]
CD133-SAL-NP	CD133^+^ ovarian cancer stem cells and nude mice bearing ovarian cancer xenografts	↑	[182]
Poly (lactic-co-glycolic acid) (PLGA) nanoparticles	Pancreatic cancer	Blocked tumor growth by 52% compared to the control.	[183]
rGO-Ag	Human ovarian cancer stem cells	↑	[184]
EGFR-SNPs	Osteosarcoma and cancer stem cells	↑	[185]
CD20-SA-NPs	Human CD20+ melanoma stem cells	↑	[186]
Salinomycin-NPs + gefitinib-NPs	Lung cancer and lung cancer stem cells	↑	[187]
Sali-NP-HER2	HER2-positive breast cancer stem cells and cancer cells	↑	[188]
Salinomycin-NPs + docetaxel-NPs	Gastric cancer cells and cancer stem cells	↑	[189]
SDLN	Liver cancer cells and cancer stem cells	↑	[190]
iTEP-Sali-ABA NP + iTEP NP-delivered paclitaxel	Metastases of 4T1 orthotopic breast tumors	↑	[191]
Salinomycin-NPs + Paclitaxell-NPs	Breast cancer stem cells and cancer cells	↑	[192]
P80-SAL-PLGA	Glioblastoma	↑	[193]
CESN	Hepatocellular carcinoma	↑	[115]
Ap-SAL-NP	Osteosarcoma cancer stem cells	↑	[118]

* CESP = salinomycin-entrapped lipid-polymer nanoparticles labeled with CD133 and EGFR aptamers; CD133-SAL-NP = salinomycin-loaded poly(lactic-co-glycolic acid)-poly(ethylene glycol) nanoparticles conjugated with CD133 antibodies; rGO-Ag = reduced graphene oxide-silver nanoparticle nanocomposites; EGFR-SNPs = EGFR aptamer-conjugated salinomycin-loaded polymer-lipid hybrid nanoparticles; CD20-SA-NPs = salinomycin-loaded lipid-polymer nanoparticles with anti-CD20 aptamer; Sali-NP-HER2 = salinomycin-loaded polymer-lipid hybrid anti-HER2 nanoparticles; SDLN = salinomycin and doxorubicin nanoliposomes; iTEP-Sali-ABA NP = immune-tolerant, elastin-like polypeptide (iTEP)-based nanoparticle; P80-SAL-PLGA = Salinomycin-encapsulated polysorbate 80-coated poly(lactic-co-glycolic acid) nanoparticles; CESN = salinomycin-loaded poly(lactic-co-glycolic acid) nanoparticles conjugated with both CD133 aptamers A15 and EGFR aptamers CL4; Ap-SAL-NP = salinomycin-loaded PEGylated poly(lactic-co-glycolic acid) nanoparticles (SAL-NP) conjugated with CD133 aptamers.
